# Highly Thiolated Poly (Beta-Amino Ester) Nanoparticles for Acute Redox Applications

**DOI:** 10.3390/gels4040080

**Published:** 2018-10-08

**Authors:** Andrew L. Lakes, David A. Puleo, J. Zach Hilt, Thomas D. Dziubla

**Affiliations:** 1 Department of Chemical and Materials Engineering, University of Kentucky, Lexington, KY 40506, USA; andrewllakes@gmail.com (A.L.L.); hilt@engr.uky.edu (J.Z.H.); 2 Department of Biomedical Engineering, University of Kentucky, Lexington, KY 40506, USA; dave.puleo@uky.edu

**Keywords:** poly (β-amino ester) (PBAE), polymeric nanoparticle, thiol, drug delivery, redox

## Abstract

Disulfides are used extensively in reversible cross-linking because of the ease of reduction into click-reactive thiols. However, the free-radical scavenging properties upon reduction are often under-considered. The free thiols produced upon reduction of this disulfide material mimic the cellular reducing chemistry (glutathione) that serves as a buffer against acute oxidative stress. A nanoparticle formulation producing biologically relevant concentrations of thiols may not only provide ample chemical conjugation sites, but potentially be useful against severe acute oxidative stress exposure, such as in targeted radioprotection. In this work, we describe the synthesis and characterization of highly thiolated poly (β-amino ester) (PBAE) nanoparticles formed from the reduction of bulk disulfide cross-linked PBAE hydrogels. Degradation-tunable PBAE hydrogels were initially synthesized containing up to 26 wt % cystamine, which were reduced into soluble thiolated oligomers and formulated into nanoparticles upon single emulsion. These thiolated nanoparticles were size-stable in phosphate buffered saline consisting of up to 11.0 ± 1.1 mM (3.7 ± 0.3 mmol thiol/g, *n* = 3 M ± SD), which is an antioxidant concentration within the order of magnitude of cellular glutathione (1–10 mM).

## 1. Introduction

Cellular oxidation is a key pathway in a wide variety of diseases, from atherosclerosis and neurodegeneration, to chemical and radiation injury [1,2]. However, cellular oxidation is also an essential component of many cellular functions. For instance, superoxide radicals are formed in the mitochondrial electron transport chain. This mitochondrial process utilizes oxygen within protein complexes and cytochrome oxidases towards ATP production, which is due to reduction of molecular oxygen [3], or in the production of respiratory bursts in neutrophils via NADPH oxidase [4]. These oxidative processes are normally held in check by endogenous reducing agents/antioxidants produced from either essential amino acid building blocks, enzymatic antioxidants, or by other materials ingested through diet such as vitamin E based tocopherols and tocotrienols [5]. A similar paradox exists with cellular reduction processes; antioxidants may also be detrimental at high levels, such as in hypervitaminosis E or with the mediation of inflammation in sepsis therapy [6,7]. It is apparent that, like most tightly regulated homeostatic systems, a fine balance exists for redox molecules between wellness and disorder [8]. As such, to treat oxidative stress disorders, this redox balance may be supplemented through selective, targeted drug delivery [9]. One approach is to supplement essential molecules already present in the cellular reactive oxygen species (ROS) defense system, including glutathione, N-acetyl cysteine (NAC), vitamins A and E, catalase, superoxide dismutase, or plant-derived polyphenols like curcumin and quercetin.

Intracellularly, glutathione (GSH) is the most abundant cytosolic small biomolecule with concentrations of 1–10 mM, depending on location/organelle [10,11]. The thiol group on GSH participates in numerous redox reactions as a reducing agent. These reactions include direct electron donation to oxidized small molecules, or via enzymes such as the irreversible glutathione S-transferase reaction to inhibit toxicity inherent with oxidized proteins and lipids [12]. Through these direct antioxidant functions, as well as indirectly through regeneration of other various antioxidant enzymes, such as glutaredoxin and glutathione peroxidase, GSH is largely oxidized into glutathione disulfide (GSSG) [13]. GSH regeneration may be performed through glutathione reductase at the expense of NADPH oxidizing to NADP+. Under normal conditions, the cytosolic GSH:GSSG ratio is near 100:1, establishing the cytosolic reducing environment [14,15]. However, under stress, the ratio may drop to below 10:1, allowing for oxidation-mediated damage of vital biomolecules, loss of mitochondrial potential, or apoptosis [12,16,17]. 

It is clear that specific local delivery to regions of the body and/or certain cell types is necessary for positive outcomes in complex oxidative stress diseases [18]. To target regions otherwise non-bioavailable or only accessible through direct local injection, active targeting strategies of systemically administered drugs/complexes have been widely studied through the use of monoclonal antibodies, ligands, aptamers, etc. [19]. Polymeric nanoparticles are a promising field of study to achieve local delivery of bioactive molecules to the site of interest, while minimizing non-specific interactions. Readily functionalizable nanoparticles are a powerful asset for covalent addition of active targeting biomolecules, such as biomolecules involved in thiol/disulfide interchange reactions for redox responsive drug release [20,21,22,23].

With the intention of supplementing the glutathione redox found in the body, our group has previously synthesized a highly biocompatible poly (β-amino ester) (PBAE) containing a disulfide cross-linker with di-primary amine functionality via cystamine (CA) [24]. These polymer gels were doped with the non-bioactive di-primary amine 4,7,10-trioxa-1,13-tridecanediamine (TTD), allowing for variation of CA content. Disulfide-state hydrogels retained their bulk form, while post-reduction released thiolated degradation products over a period of 2–3 days. An increase in CA content enhanced cellular viability of degradation products in the thiolated form, but showed no change in the disulfide form. Furthermore, the thiolated degradation products were able to protect endothelial cells from oxidative damage. While we could form up to 75 mol % CA of the total amines (75/25 mol % CA/TTD), it was previously not possible to synthesize a hydrogel with CA accounting for 100 mol % of the amine used because of CA solubility limits in dimethylsulfoxide (DMSO) [24]. Previously, we found that when these hydrogels contained greater than 66 mol % of the amine of CA cross-linker, they would solubilize completely in reducing conditions because of the breakdown of disulfide linkages into shorter chain oligomers. In this work, we have modified the reaction conditions in order to reach 100 mol % CA of the amines through the use of a non-nucleophilic Michael addition catalyst, 1,8-diazabicycloundec-7-ene (DBU). With DBU utilized to increase CA reactivity, two systems were made with 100 mol % CA, either using diethylene glycol diacrylate (DEGDA) or the more hydrophobic 1,6-hexanediol ethoxylate diacrylate (HEDA) at a 1:1 total acrylate to total amine ratio (stoichiometric based on reactive sites). Using thiolated oligomers produced via reduction of 100 mol % CA of amine hydrogels, these oligomers form stable nanoparticles upon single emulsion of which we discover the material properties and function as a conjugatable thiol delivery vehicle for potential redox applications.

## 2. Results

### 2.1. Cystamine Hydrogel Material Characteristics

**Figure 1:**
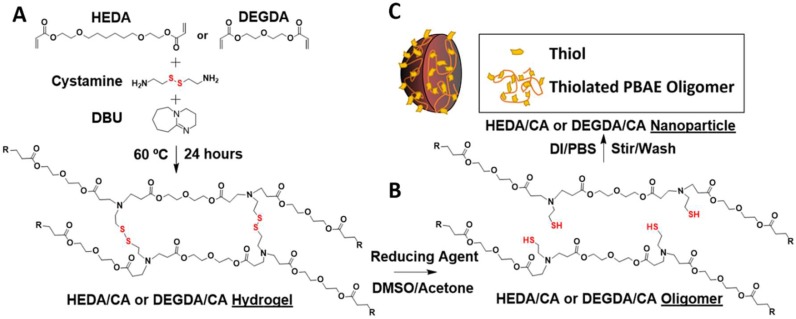
Hydrogel-to-nanoparticle synthesis scheme. (**A**) disulfide poly (β-amino ester) (PBAE) hydrogel and conversion into (**B**) thiolated oligomers via a reducing agent (2-mercaptoethanol), and (**C**) single-emulsion into thiolated nanoparticles.

**Figure 2:**
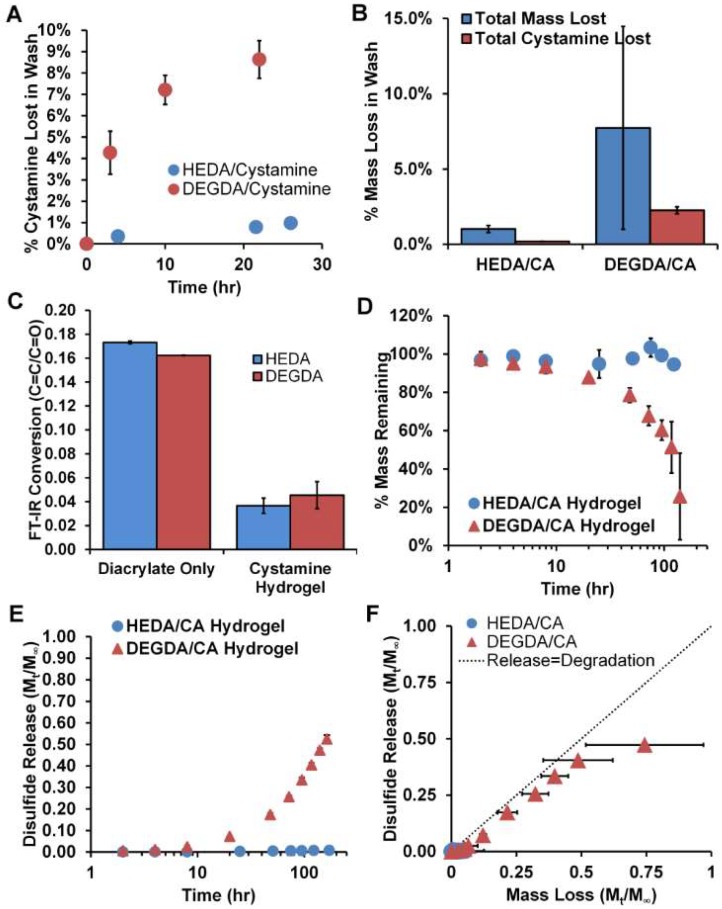
Disulfide hydrogel characteristics. (**A**) % CA loss of total CA added after washing in dimethylsulfoxide (DMSO), (**B**) % mass loss of hydrogel CA based on total mass before and after wash, and (**C**) conversion with FT-IR. (**D**) % mass remaining during sink condition degradation at 37 °C, (**E**) disulfide release, and (**F**) comparison of disulfide release to mass loss over time. *n* = 3, M ± SD.

In these PBAE reactions, aza-type Michael addition requires the amine species to act as a nucleophile to acrylate groups for the reaction to proceed. Through the addition of DBU [25], a non-nucleophilic base with a pKa greater than that of CA (pKa CA = 9.91, 9.3; pKa DBU = 11.3, 5.68), the CA primary amines remain deprotonated for a large extent of the reaction. Utilizing DBU, we produced gels containing 100 mol % CA of the amine hydrogels, which were previously unattainable (Figure 1A). Using this approach, two types of hydrogels were formed at a ratio of acrylate to amine of 1:1, HEDA/CA, and DEGDA/CA. These two diacrylates were chosen because of their difference in hydrophilicity, which could hypothetically alter the PBAE degradation rates. Where both DEGDA and HEDA contain two glycol groups, HEDA differs in that the glycol groups are separated by a six carbon aliphatic chain. The 100 mol % CA of the amine was added to the reactants for a total of 20 wt % for HEDA and 26% for the DEGDA preparations. After polymerization, the samples were washed using DMSO to remove unreacted and soluble components and the DBU catalyst, and analyzed for CA loss (Figure 2A) and total mass loss (Figure 2B). While the HEDA/CA hydrogel had a 1.0 ± 0.24% total mass loss with 0.2 ± 0.01% of that from the CA alone, the DEGDA/CA hydrogel possessed a 7.7 ± 6.7% total mass loss with 2.3 ± 0.23% loss being from CA, *n* = 3, mean ± standard deviation. Washing also de-convoluted the Fourier Transform Infrared Spectroscopy (FT-IR) spectra from DBU’s protonated C=N form with the C=C peak from the diacrylate, allowing us to assess the C=C conversion and proving removal of DBU from the system.

After washing, freeze dried hydrogels were analyzed using FT-IR to determine the extent of reaction by comparing the diacrylate ratio of the carbon double bond to carbonyl ratio (C=C/C=O) (Figure 2C). Because of the ratio of acrylates to amines used being at 1:1, it was not expected that the C=C/C=O ratio would drop completely to 0. The HEDA/CA hydrogel had a slightly greater conversion with a raw value of 0.036 ± 0.0063 C=C/C=O (21 ± 3.7% of the C=C/C=O final/initial), where the DEGDA/CA hydrogel was found to have 0.045 ± 0.011 (28 ± 7.0% of the C=C/C=O final/initial) remaining, *n* = 3, mean ± standard deviation.

These hydrogels were theoretically predicted to show hydrolytic degradation of ester bonds in aqueous media. While the DEGDA/CA hydrogel degraded measurably over 7 days to roughly 26% ± 23% of their mass remaining (Figure 2D) with 52 ± 2% of the disulfide-containing degradation products released (*n* = 3, mean ± standard deviation), the HEDA formulation remained within an error of 100% mass remaining after 7 days, and less than 1% of the disulfide-containing degradation products released (Figure 2E). Comparing the disulfide release to polymer degradation rate (Figure 2F), disulfide release appears to be degradation controlled (i.e., close to the 45° line), if not slightly slower than degradation, perhaps because of over-reporting of degradation products as a result of small hydrogel fragments being aspirated at later time points, and thus not detected in the solution for Ellman’s detection assay.

### 2.2. Properties of Reduced Hydrogels

Because of the presence of disulfide cross-links in the gel backbone, reducing conditions will cleave the disulfide bonds to form a thiolated linear oligomer. Bulk hydrogel reduction proceeded for 1 h with a vigorously vortexed solution under argon until the matrix was dissolved, using 50/50 vol % of 20:1 stoichiometric reducing agent 2-mercaptoethanol (2-ME) in acetone or DMSO. Following reduction, hydrogels were solubilized in either DMSO or acetone, whereupon they were analyzed using mass spectrometry. With the intention of detecting differences in oligomeric molecular weight incorporation into the nanoparticles, a second set of oligomers was also analyzed for molecular weight *after* nanoparticle formation, which had been washed, freeze dried, and re-reduced with 2-ME in acetone to retain the initial oligomeric form. The median molecular weight found for the reduced HEDA/CA hydrogel was 2000 m/z before nanoparticle formation and 2600 m/z after nanoparticle formation (Figure S1A). However, for the reduced DEGDA/CA hydrogel, the median molecular weight was 2500 m/z before and after nanoparticle formation, yet some smaller m/z fragments are seen to be diminished in intensity (Figure S1B). For a molecular weight of 2500 Da, approximately 10-mers are the median size of the reduced hydrogel products. More dramatic, however, was the increased higher molecular weight tail for the reduced materials after nanoparticle formation and washing. Because of the low ionization intensity of MALDI MS, it is typical to assume that *z* = 1 for these polymers.

### 2.3. Thiolated Nanoparticles

**Figure 3:**
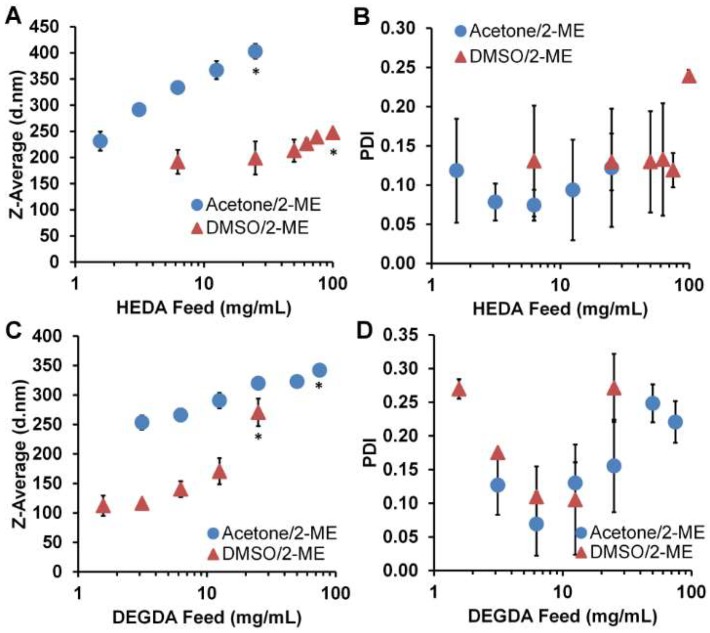
Comparison of oligomer feed concentration. (**A,C**) Comparison of nanoparticle *z*-average diameter and (**B,D**) polydispersity index of HEDA/CA and DEGDA/CA based thiolated nanoparticles, respectively, in phosphate-buffered saline/polyvinyl alcohol/ethylenediaminetetraacetic acid (PBS/PVA/EDTA). *n* = 3, M ± SD. * Maximum concentration before particles became unstable.

Thiolated PBAE nanoparticles were formed through a single step solvent/anti-solvent formulation strategy using reduced oligomers (Figure 1B,C). After adding hydrogels into organic solvent in the presence of a reducing agent, the thiolated oligomers formed were then added dropwise into an aqueous anti-solvent of phosphate-buffered saline (PBS) containing 1 wt % polyvinyl alcohol (PVA) for stabilization and 10 mM ethylenediaminetetraacetic acid (EDTA) to inhibit thiol oxidation from cations. Particle sizes were found to be dependent upon both feed concentration and organic solvent selection (DMSO or acetone). For both the HEDA/CA and DEGDA/CA systems, acetone use resulted in larger *z*-average particle diameters, which increased with an increase in feed concentration (Figure 3). With the HEDA/CA formulation, the maximum particle feed concentration was found to be 100 mg/mL for the DMSO solvent, and 25 mg/mL for acetone for the nanoparticles to remain stable upon synthesis (Figure 3A,B). For DEGDA/CA, nanoparticles were unstable at concentrations exceeding 75 mg/mL in acetone, and 25 mg/mL for DMSO (Figure 3C,D).

For electron microscopy imaging, we used deionized (DI) water instead of PBS to form reduced HEDA/CA nanoparticles. Particles formed in DI water possessed a *z*-average diameter of 150 nm with a polydispersity index (PDI) of 0.150 from dynamic light scattering (DLS) readings (Figure S2F). After washing, scanning electron microscopy (SEM) images were obtained. Interestingly, we found that SEM samples prepared at a high concentration (4 mg/mL used) facilitated film formation upon the droplet drying onto the aluminum foil substrate (Figure S2A–C). Energy dispersive X-ray spectroscopy (EDS) elemental analysis showed the film to contain 2.2% sulfur and 79.5% carbon (Figure S2D). At a low particle concentration (0.01 mg/mL), however, particles appeared to retain their solution morphology and were found to be in the correct size range as DLS measurements, albeit lightly agglomerated (Figure S2E). 

#### 2.3.1. Characterization of Thiol Functionality

In order to visualize the thiolated nanoparticles, we reacted HEDA/CA and DEGDA/CA nanoparticles with a thiol-reactive fluorescent maleimide, N-(1-pyrenyl)maleimide (NPM), in DMSO. Dynamic light scattering of particle sizes was found to be 375 nm with 50% DMSO vs. 150 nm with 8% DMSO, where the addition of NPM (with or without) did not affect particle sizes using 50% DMSO (Figures S2F and S3A). Representative images of visible nanoparticle aggregates were taken with a fluorescent microscope using an ultraviolet filter or in bright field, before and after NPM addition, and with either HEDA/CA or DEGDA/CA nanoparticles (Figure S3B). It was seen that both the HEDA/CA and DEGDA/CA nanoparticles/aggregates fluoresced with the addition of NPM, but not without, indicating thiol functionality.

#### 2.3.2. Nanoparticle Kinetics

**Figure 4:**
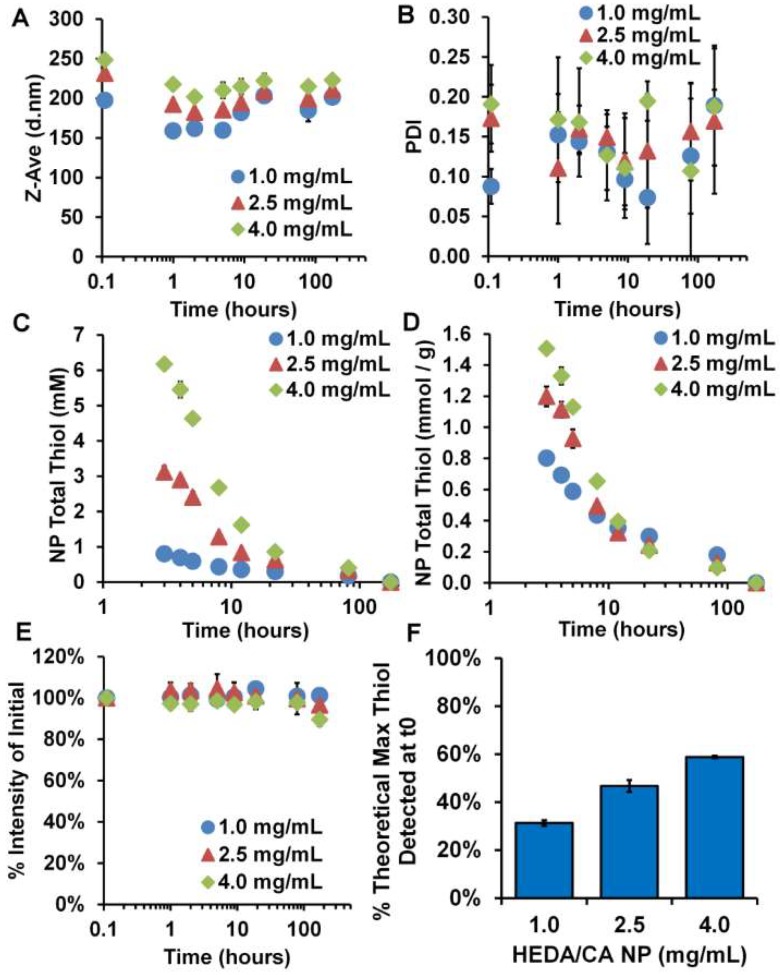
Kinetics plots of HEDA/CA nanoparticles in PBS/EDTA/EDTA. (**A**) *z*-average diameter, (**B**) polydispersity index (PDI), (**C**) total thiol found, (**D**) total thiol found per mass of nanoparticles, (**E**) % intensity of dispersion over time, and (**F**) % of the theoretical maximum thiol concentration found after washing. *n* = 3, M ± SD.

Activity, size, and degradation kinetics were measured with nanoparticles formed in PBS/PVA/EDTA, under 37 °C incubation at varying concentrations. Concentrations were found via freeze drying and weighing nanoparticle pellets. To compare the ideal nanoparticle synthesis conditions for each system, HEDA/CA nanoparticles were formed with DMSO, and DEGDA nanoparticles were formed with acetone. The HEDA/CA particles possessed sizes from 200–250 nm with PDI between 0.1–0.2 (Figure 4A,B). Similar to the bulk hydrogel, there was no significant evidence of degradation occurring, as monitored by DLS intensity over time (Figure 4E). 

Ellman’s thiol assay was performed on the nanoparticle bulk solution at each time point without washing to include both nanoparticle bound thiols and any degradation products. Thiols were found to be present on the particles with Ellman’s assay up to 7 days (Figure 4C,D), with the maximum specific thiol release (mmol/g nanoparticles) at the highest particle concentration (4.0 mg/mL) depending on the formulation. These initial synthesis concentrations were taken as soon as possible after the 3 wash steps were complete, which equates to 3 h from initial formation where 31%, 47%, and 59% of the theoretical maximum thiols were detected (Figure 4F). The decrease in specific thiol content and recovery was likely due to the kinetics of thiol re-oxidation being concentration dependent. 

**Figure 5:**
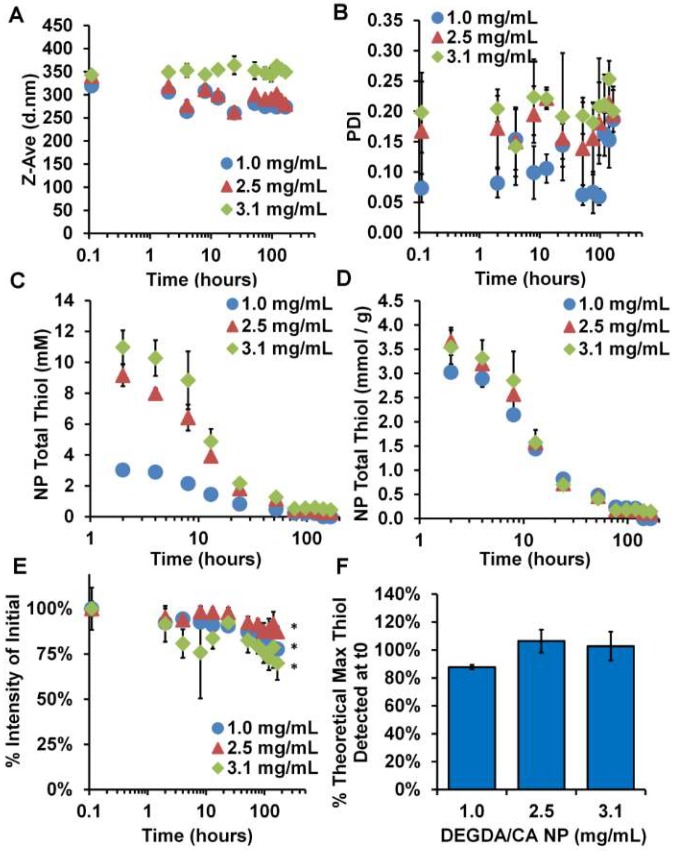
Size and activity kinetics of DEGDA/CA nanoparticles in PBS/PVA/EDTA. (**A**) *z*-average diameter, (**B**) polydispersity index, (**C**) total thiol found, (**D**) total thiol found per mass of nanoparticles, (**E**) % intensity of dispersion over time, and (**F**) % of the theoretical maximum thiol concentration found after washing. *n* = 3, M ± SD. * Indicates *p* < 0.05 intensity change from original.

Comparing the DEGDA/CA nanoparticles to the HEDA/CA nanoparticles, the presence of thiols also lasted over 7 days, as beyond that minimal levels were being detected (Figure 5C,D). However, the starting concentration was much closer to the theoretical maximum (Figure 5F) at averages of 88 ± 1.5, 106 ± 8.2, and 103 ± 10.2% of the theoretical maximum, and the lower concentration nanoparticle system showed the lowest specific thiol content (mmol/g nanoparticles). In contrast to the HEDA/CA system where the bulk hydrogel and nanoparticles showed no degradation, the DEGDA/CA nanoparticle system showed signs of degradation to compliment the bulk hydrogel degradation. While the *z*-average particle sizes did not change consistently across the concentrations starting between 300–350 nm with a PDI of 0.075–0.20 (Figure 5A,B), the % intensity of the original were statistically different after 7 days of degradation, decreasing by 13–30% from the original (Figure 5E).

## 3. Discussion

We have formulated a gel-to-nanoparticle production system in which high disulfide-containing PBAE hydrogels are reduced into oligomers in organic solvent, and precipitated via single emulsion into an aqueous anti-solvent to produce highly thiolated nanoparticles. With this two-phase technique, the bulk material of disulfide hydrogels may be stored in a non-volatile, oxidized state prior to nanoparticle formulation, circumventing difficult storage conditions, and allowing fast and cost-effective nanoparticle synthesis for immediate use. 

While disulfide bridges are often found in nanoparticles, they are more often utilized for instance as redox-sensitive cross-linkers that dissolve for drug release upon reduction [26,27,28,29,30,31], for extended circulation formulations [32], with thiol-ene type reactions as a reactive cross-linker [33,34], or to reduce disulfides on antibodies for antibody/nanoparticle surface conjugation [35]. For the purpose of antioxidant delivery, however, nanoparticles delivered in the thiol state are not as widely reported compared to thiol-based small molecule systems, perhaps because of the rapid oxidation rates in aqueous solution [36]. Where other antioxidant systems may show antioxidant drug release through fast (24–48 h) [37] or slow (weeks) [38] degradative processes, this gel-like nanoparticle system is different in that maximal antioxidant capacity is available at the onset of treatment, with single log reduction over ~48 h (Figure 5). Compared to similar applications in the literature [28,36], our particles were highly thiolated at up to 12 wt % (3.7 ± 0.28, and 1.5 ± 0.01 mmol SH/g nanoparticles for the DEGDA/CA formulation and HEDA/CA formulation, respectively) (Figure 4D and Figure 5D). For applications of antioxidant delivery, organ protection prior to radiotherapy, or post oxidation event, such as ischemia-reperfusion injury, a short but potent thiol half-life formulation with high initial activity may be desirable. Furthermore, this high drug loading was intrinsic to the material via use of the inexpensive cross-linker, CA, without requirement of commonly used and less economical cross-linkers such as SH-PEG-SH or succinimidyl 3-(2-pyridyldithio)propionate) (SPDP). 

Cystamine cross-linked hydrogels with high disulfide content (utilized above the threshold disulfide-content to dissolve in the presence of a reducing agent in organic solvents [24]) allowed for precipitation of hydrogel oligomers in aqueous systems. Even though it is thought that both the DEGDA/CA and HEDA/CA hydrogels were synthesized as PBAEs through Michael addition of the acrylates and amines to form ester linkages, only the DEGDA bulk hydrogel system showed a measurable degradation rate and disulfide release over 7 days (Figure 2D,E). HEDA is more hydrophobic than DEGDA in that it contains an aliphatic 6 carbon chain compared to DEGDA with 2 carbons, and therefore may inhibit hydrolysis of the ester bonds, perhaps to the extent of being non-degradable in aqueous, non-biological environments. Hydrophobic effects due to PBAE constituents have been shown in the literature to greatly affect degradation time. For instance, Hawkins et al. used a similar PBAE hydrogel utilizing DEGDA and the primary amine isobutylamine instead of the hydrophilic di-primary amine cystamine to form hydrogels upon free radical polymerization of acrylate endcaps, with a resulting degradation period of 4 months, considerably longer than our hydrogels period of 8 days [39]. Swelling studies comparing the two hydrogel systems could aid in this hypothesis of decreased polymer-solvent interaction with HEDA/CA hydrogels [24]. The extent of reaction as measured by FT-IR was only slightly greater for the HEDA/CA compared to DEGDA/CA, but the increased extent of reaction is unlikely to explain the non-degradability of the material. These conversion numbers found are also similar to those recorded in our previous studies with the TTD-doped DEGDA/CA hydrogels, at 0.04 C=C/C=O, which showed fast hydrolysis in water [24]. 

Upon reduction of PBAE hydrogels containing CA, mass spectrometry showed that lower molecular weight fragments were washed out after nanoparticle formation for the HEDA/CA oligomer/nanoparticle system, compared to before washing. As not all oligomers formed upon hydrogel reduction would be of equal chain length, it is unlikely that all oligomers were incorporated into self-assembled nanoparticles produced as a result of amphiphilic differences. Upon centrifugal washing, the nanoparticles were separated from unincorporated oligomers in the solution, as well as separated from low molecular weight fragments which would diffuse from the nanoparticle during centrifugation, unlike larger fragments which would show diffusional limitations. As a result of the stoichiometric excess of 2-ME used to reduce both the hydrogel and nanoparticles, this result cannot be explained by re-oxidation of oligomers. 

After reduction of the hydrogels into thiolated oligomers, the nanoparticles formed remained stable for over 8 days without aggregation in solution, even upon complete oxidation of thiols after 7 days. This stability was likely enhanced by the addition of PVA, and initial thiol activity was extended with the presence of EDTA to inhibit the effects of metal cations, which are known to increase thiol oxidation rate [40]. Because of the similarity in hydrogel composition to that of Lakes et al. [24], it is thought that the final degradation products of these oligomeric chains is similar, with the major species produced from ester hydrolysis into soluble species mostly <300 MW. Although not tested in this work, a serum-containing media may lower thiol activity through thiol-disulfide exchange in proteins. It is also possible that in an intracellular environment which has high reducing potential, bound-thiols may persist longer and aid in glutathione/cysteine antioxidant capacity in times of oxidative stress. Future experiments using a cellular environment may also increase the nanoparticle degradation rate owing to the presence of esterase to cleave the PBAE ester bonds. 

While thiolated nanoparticles did not aggregate at the solution concentrations of Figure 4A and Figure 5A, nanoparticles did demonstrate fusion and flow when allowed to dry on a surface in open air at high concentration. This was demonstrated with electron microscopy where nanoparticles formed a film, likely triggered by re-oxidation of the high concentration of thiols present. Where typical particle aggregation may show particle–particle interaction under SEM, the polymers were likely above the T_g_, if not also the T_m_ because of the film formation at high concentration, and individual soft spheres at low concentration. It is hypothesized that from the combination of capillary forces and droplet air drying, the higher concentration polymer on the droplet edge was from re-oxidation of the polymer into a concentrated film. We attempted to run DSC on the reduced polymer to verify if the thiolated nanoparticles were in a state above the T_m_ at room temperature, but we were unable to maintain the polymer in the reduced form while adequately removing solvent and reducing agents without inducing oxidation. As solid nanoparticles are typically less prone to undesired effects such as aggregation, future work to increase the T_g_ would be desirable, perhaps through increasing the oligomeric chain molecular weight or via crosslinking.

The increase in nanoparticle size based upon feed concentration was possibly due to an intrinsic viscosity increase upon increased concentration. Similarly, the size dependence upon solvent variation was likely due to Flory-Huggins derived solvent miscibility differences [41]. While fluorescent imaging of nanoparticles with the thiol-reactive dye, NPM, was not expected to show individual nanoparticles because of optical limitations, we did find fluorescence of the visible aggregates (Figure S3). Since NPM is only weakly fluorescent before maleimide reaction, this is a further implication that thiols were present on the nanoparticles as is seen with the direct kinetic Ellman’s assay. With this, it is theorized that through co-incubation of IgG type antibodies with these thiolated nanoparticles, interchain disulfides would thiol exchange with nanoparticle surface exposed thiols, creating the potential for targeted antioxidant delivery.

Although delivery of antioxidants may superficially appear a straightforward process towards cellular protection and oxidative disease prevention through sequestration of free radicals, prior research has shown that antioxidant delivery has strict requirements upon dosage, targeting, and duration of delivery to create desirable cellular interactions. For instance, delivery of NAC, one of three ingredients for glutathione synthesis, has clinical applications for the treatment of acetaminophen overdose [42]. This is through competitive binding of the acetaminophen metabolite N-acetyl-p-benzoquinone imine, blocking glutathione depletion. However, NAC delivery has also been shown to interrupt p53 pathways, a multifaceted protein which can initiate apoptosis and a key tumor suppressor implicated in DNA repair, hypoxia, and oncogene activation [43,44]. In mice exposed to inhaled *Cre* recombinase adenovirus, which have an increased propensity for lung cancer, it was found that NAC or vitamin E supplements increased cancer progression rates. When *Trp53* knockout mice were used, there was no effect of antioxidant administration, identifying ROS-mediated p53 apoptosis inhibition as a likely mechanism for antioxidant stimulated increase in cancer progression [45]. These findings suggest there is a risk of non-targeted antioxidant delivery to those already with cancer, especially to high-risk individuals such as smokers, but do not directly identify changes in tumor initiation or prevention. With this strict necessity for targeted delivery in mind, we sought an antioxidant nanoparticle system which could be readily conjugated for targeted acute antioxidant applications requiring high initial antioxidant capacity. Furthermore, as a result of the quick oxidation kinetics of thiols, we sought to form these nanoparticles from a stable material.

## 4. Conclusions

Through reduction of disulfide containing hydrogels of high drug content, we formulated and characterized thiol-bound nanoparticles directly from solid hydrogels, which showed tunable degradability, size, and activity. The nanoparticles formed were shown to be highly thiol-containing, corroborated via Ellman’s assay and reaction with fluorescent NPM, demonstrating functionality of the thiol groups. As prior research has shown, these thiol groups are highly biologically relevant, may aid in processes such as reduction or prophylaxis of pathophysiological oxidative stress, and be useful as conjugate moieties found at high concentrations using inexpensive starting materials.

## 5. Materials and Methods

### 5.1. Materials

All reagents were used as received without further purification steps. Cystamine dihydrochloride (CA), N-(1-pyrenyl)maleimide (NPM), 1,8-Diazabicycloundec-7-ene (DBU), 5,5’-dithiobis(2-nitrobenzoic acid) (DTNB/Ellman’s Reagent), 1,6-hexanediol ethoxylate diacrylate (HEDA), and 2-mercaptoethanol (2-ME) were purchased from Sigma-Aldrich (St. Louis, MO, USA). Diethylene glycol diacrylate (DEGDA) was purchased from Polysciences Inc. (Warrington, PA, USA). Immobilized tris(2-carboxyethyl)phosphine (TCEP) beads were purchased through Thermo Scientific (Waltham, MA, USA). All solvents were purchased through either Fisher Scientific (Waltham, MA, USA) or Pharmco-AAPER (Brookfield, CT, USA).

### 5.2. Cystamine Hydrogel Synthesis and Washing

CA was first dissolved at 185 mg/mL into a 25/75 vol % mixture of DBU catalyst/DMSO, respectively. Because CA is near the solubility limit in the DBU/DMSO solution, CA was added to the solvent during vortexing and then immediately immersed in a sonication bath. Then, diacrylate (either HEDA or DEGDA) at a stoichiometric ratio between acrylate and amine reactive sites (33 mol % CA and 67 mol % diacrylate), was added by mass and vortexed briefly. The reaction contents were then dispensed onto a casting ring (Teflon on glass with a weighted cylindrical ring, open top) for 24 h at 60 °C in a large oven. 

Hydrogel washing post-synthesis was performed for quantification of unreacted CA and diacrylate. Washing was performed three times using 10× excess volume of anhydrous DMSO by mass with 4 Å molecular sieves in an argon atmosphere for three time periods (1 h, 4 h, 24 h) with the supernatant collected to measure CA loss, and DMSO replaced. The disulfide-containing supernatant was then reduced with immobilized TCEP beads for material loss analysis with Ellman’s thiol detection assay. Washing was deemed complete when there was <1 mM disulfide detected in the final wash volume (<1.5% free CA). After DMSO washing, DMSO was removed from the gels using acetone extraction (10× volume) three times over 8 h. Since CA is not soluble in acetone, we did not collect acetone fractions for CA detection. The hydrogels were then allowed to air dry for 1 h, followed by freeze drying for 24 h whereupon a change in mass was measured for based on the original mass of ingredients added. 

### 5.3. Extent of Hydrogel Conversion

Fourier Transform Infrared Spectroscopy (FT-IR) (Varian 7000e, Agilent, Santa Clara, CA, USA) was used to determine extent of reaction by tracking carbon double bond to carbonyl ratio on the acrylates before and after reaction (C=C/C=O ester ratio). Peak areas were measured using Varian Resolutions Pro software. Samples were prepared to react directly upon a 60 °C heated ATR crystal to mimic casting ring and oven conditions at the start of the measurement. 

### 5.4. Bulk Hydrogel Degradation and Disulfide Release

Hydrogels were sectioned into <33 mg discs, weighed, and added into weighed 2 mL microcentrifuge tubes. Using 10 mM PBS at pH 7.4, hydrogels were degraded at 16.6 mg/mL at 37 °C. PBS supernatant was immediately analyzed for disulfide release. The released disulfide containing degradation products were first reduced using immobilized TCEP beads and then measured for thiol content using Ellman’s assay. For change in mass time points, hydrogels were removed from the PBS, freeze dried 24 h, and weighed. 

### 5.5. Nanoparticle Synthesis

Hydrogels were reduced with 2-ME and organic solvent (acetone or DMSO) at 20× molar excess and vortexed vigorously under argon atmosphere to solubilize the hydrogels into thiolated linear chain PBAE oligomer over 30 min. This oligomeric solution was either used as is or diluted with acetone or DMSO, and then added dropwise to an aqueous anti-solvent (DI or PBS, with or without PVA) to precipitate the single emulsion nanoparticles under mild vortexing. With a final 8% v/v organic concentration after initial single emulsion nanoparticle formation, nanoparticles were washed three times under appropriate centrifugation periods for 40 min at 30,000 g to remove organic solvent, reducing agent, and uncoupled oligomers.

### 5.6. Mass Spectrometry

Mass spectrometry data were obtained at the University of Kentucky Mass Spectrometry Facility using a Bruker Ultraflextreme time-of-flight mass spectrometer equipped with a smartbeam-II solid state laser (Nd:YAG, 355 nm), using dihydroxybenzoic acid as the matrix for matrix-assisted laser desorption/ionization (MALDI) sample preparation in linear mode. Two sample types of reduced hydrogels were analyzed, (1) hydrogels reduced with 2-ME in acetone, and (2) hydrogels reduced with 2-ME in acetone, formed into nanoparticle in DI water which were washed, and then the solution freeze dried and reduced again using 2-ME and acetone.

### 5.7. Microscopy

Electron microscopy was performed at the University of Kentucky’s Electron Microscopy Center. SEM was first performed with a Hitachi S4300 (Hitachi, Schaumburg, IL, USA) with a low concentration sample (0.01 mg/mL) of HEDA/CA nanoparticles formed in DI water which were washed, flash frozen, and freeze dried onto carbon tape. A high concentration nanoparticle sample (5 mg/mL) was formed with an HEDA/CA hydrogel, which was reduced with 2-ME and formed into DI water. This sample was then washed and air dried onto aluminum foil (instead of flash frozen and freeze dried). SEM and EDS were analyzed using an FEI Helios DualBeam FIB/SEM (ThermoFisher, Waltham, MA, USA) for imaging and elemental analysis. 

For fluorescent conjugate nanoparticle imaging, the thiol-reactive fluorescent dye, NPM, was reacted at 10× molar excess with HEDA/CA and DEGDA/CA thiolated nanoparticles for 1 h in 50/50 vol % DMSO/DI water at room temperature, and then washed out 3 times. Particle sizes were taken before and after conjugation. Fluorescent microscope images were taken at 1 ms for bright field and 60 s for UV filtered images.

### 5.8. Nanoparticle Size vs. Feed Concentration

Using 2-ME reduced hydrogels in either acetone or DMSO at various concentrations, nanoparticles were added dropwise into a vortexing tube of 10 mM PBS containing 1 wt % of 78% hydrolyzed 6000 MW poly(vinyl alcohol) (PVA) and 1 mM EDTA. Size (*z*-average diameter) and polydispersity index (PDI) were determined with a Malvern Zetasizer Nano (Malvern, Westborough, MA, USA).

### 5.9. Nanoparticle Kinetic Size and Thiol Activity

Using three feed concentrations of 25, 50, and 75 mg/mL 2-ME reduced hydrogel in either DMSO (for HEDA/CA) or acetone (for DEGDA/CA), nanoparticles were formed from dropwise addition into the PBS/PVA/EDTA solution and washed 3 times. A fourth wash was collected to determine the background reducing agent levels, and also to determine pellet mass after freeze drying for final nanoparticle concentrations formed (listed in plot legends of Figure 4 and Figure 5). We chose DMSO for HEDA/CA and acetone for DEGDA/CA as they formed at the highest concentration of nanoparticles, respectively, allowing for ease of pellet mass measurement. Kinetic nanoparticle-bound thiol content was measured using a modified version of Ellman’s assay with aliquots from 37 °C incubated samples. After 20 min of room temperature incubation of nanoparticles with 5,5’-dithiobis-(2-nitrobenzoic acid) (DTNB) solution, the mixture was centrifuged for 5 min at 30,000 g to remove nanoparticle precipitates and the supernatant that contained cleaved DTNB. The resulting colorful TNB (thiobis-(2-nitrobenzoic acid) was read at 412 nm at each time point. Percent intensity of initial time point measurements were made with derived total count rate measurements from the DLS. Theoretical max thiol was calculated from knowledge of the pellet mass and hydrogel CA mass fraction, assuming an equal distribution of CA-containing oligomers remained in the nanoparticle compared to what was washed out. 

## Supplementary Materials

The following are available online at http://www.mdpi.com/2310-2861/4/4/80/s1, Figure S1: Mass spectrometry of thiolated oligomers, Figure S2: SEM of thiolated HEDA/CA nanoparticles at high concentration (4 mg/mL) forming a film at droplet boundary, and particle characteristics, Figure S3: Particle conjugation with NPM.
