# Supplementary material for: Highly Thiolated Poly (Beta-Amino Ester) Nanoparticles for Acute Redox Applications
Source: Gels. 2018 Oct 8;4(4):80. doi: 10.3390/gels4040080 (PMC6318580; doi:10.3390/gels4040080)
Supplement: Supplementary file 1 [file gels-04-00080-s001.pdf]

Highly Thiolated Poly(beta-amino ester) Nanoparticles for Acute Redox Applications

Andrew L. Lakes [1], David A. Puleo [2], J. Zach Hilt [1], Thomas D. Dziubla [1]*

[1] Department of Chemical and Materials Engineering, University of Kentucky, Lexington, KY, 40506, USA.

[2] Department of Biomedical Engineering, University of Kentucky, Lexington, KY, 40506, USA.

*Corresponding Author

E-mail: thomas.dziubla@uky.edu

Telephone: (859) 257-4063

FAX:

SUPPLEMENTARY INFORMATION

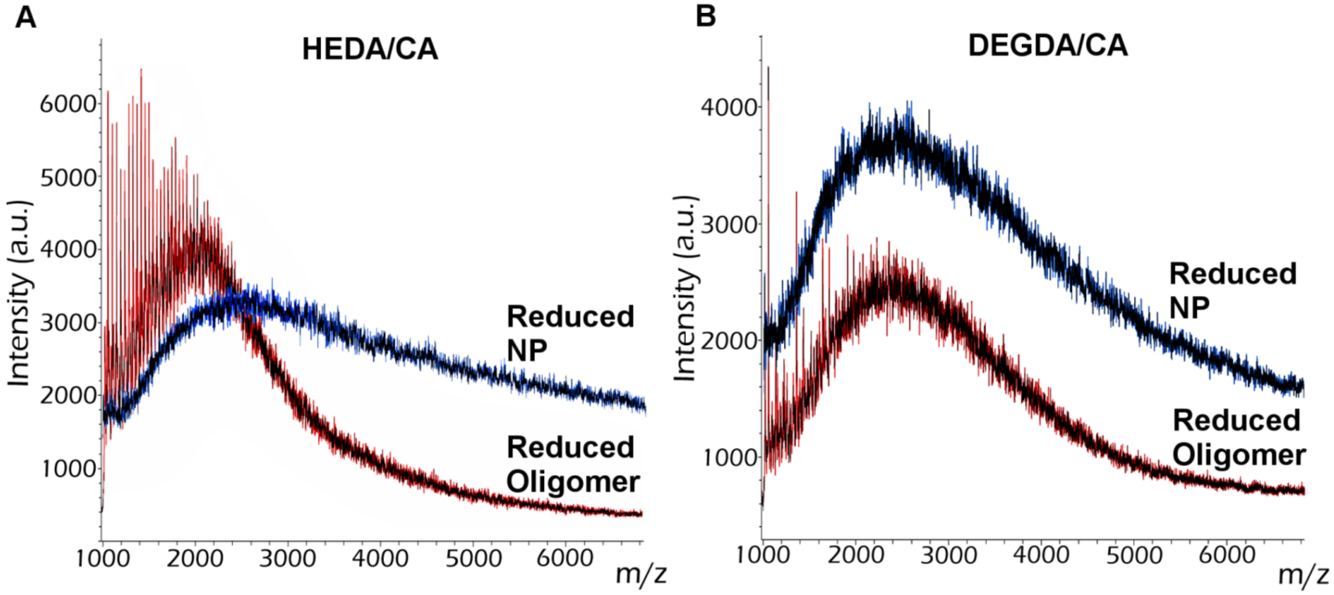

Figure S1. Mass spectrometry of thiolated oligomers. A) HEDA/CA reduced hydrogels before (red) and after (blue) nanoparticle formation. B) DEGDA/CA reduced hydrogels before (red) and after (blue) nanoparticle formation.

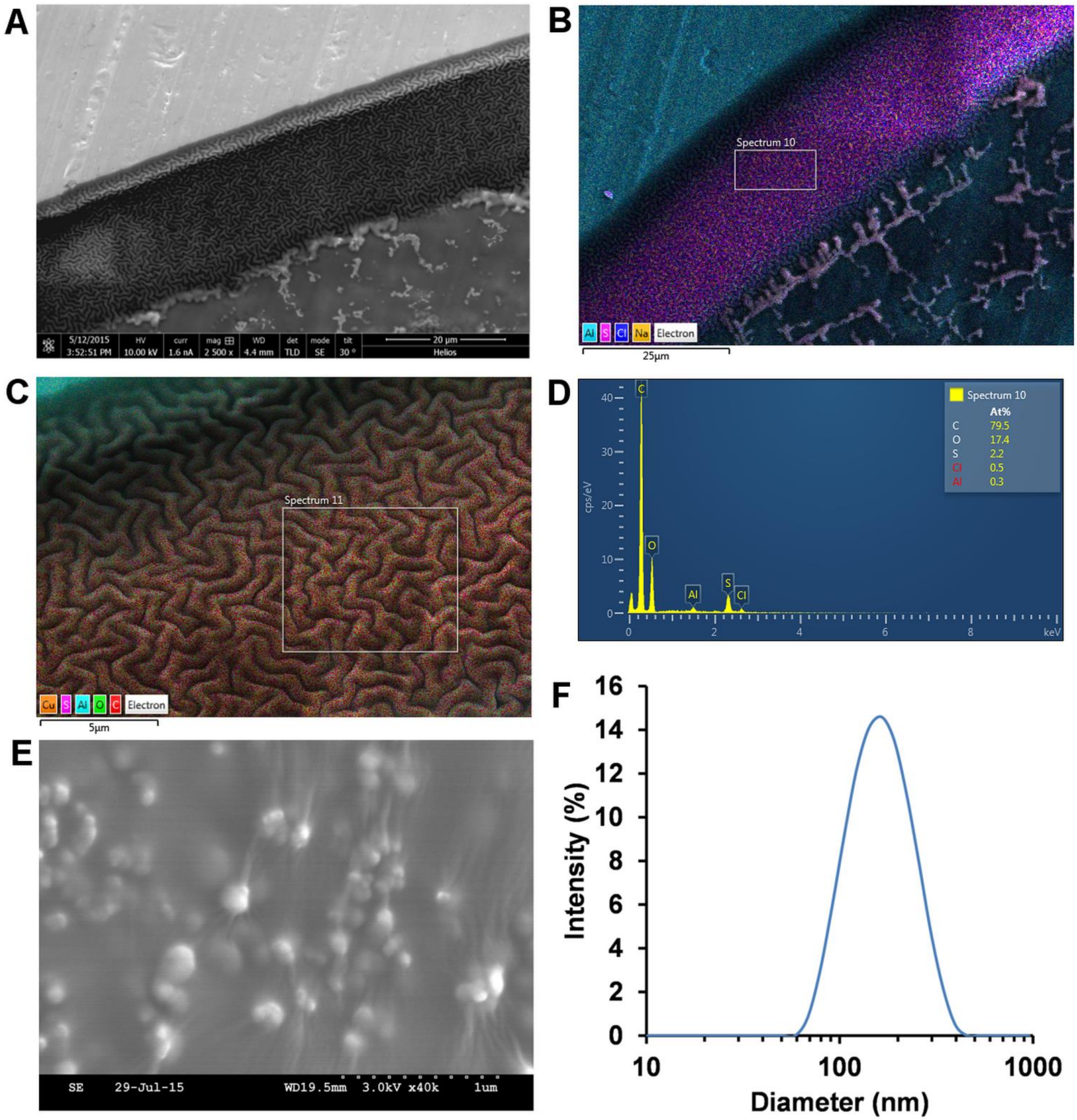

Figure S2. SEM of thiolated HEDA/CA nanoparticles at high concentration (4 mg/mL) forming a film at droplet boundary, and particle characteristics. A) Zoomed out micrograph, B) zoomed out micrograph overlaid with elemental analysis chromatogram – purple indicates sulfur from CA, blue indicates aluminum from the substrate. C) Zoomed in micrograph of film showing surface deformation upon solvent evaporation, and D) energy dispersive X-ray spectroscopy (EDS) spectrum. E) SEM of nanoparticles at low concentration (0.01 mg/mL) in DI water, and F) characteristic particle size in DI water of HEDA/CA nanoparticles via DLS.

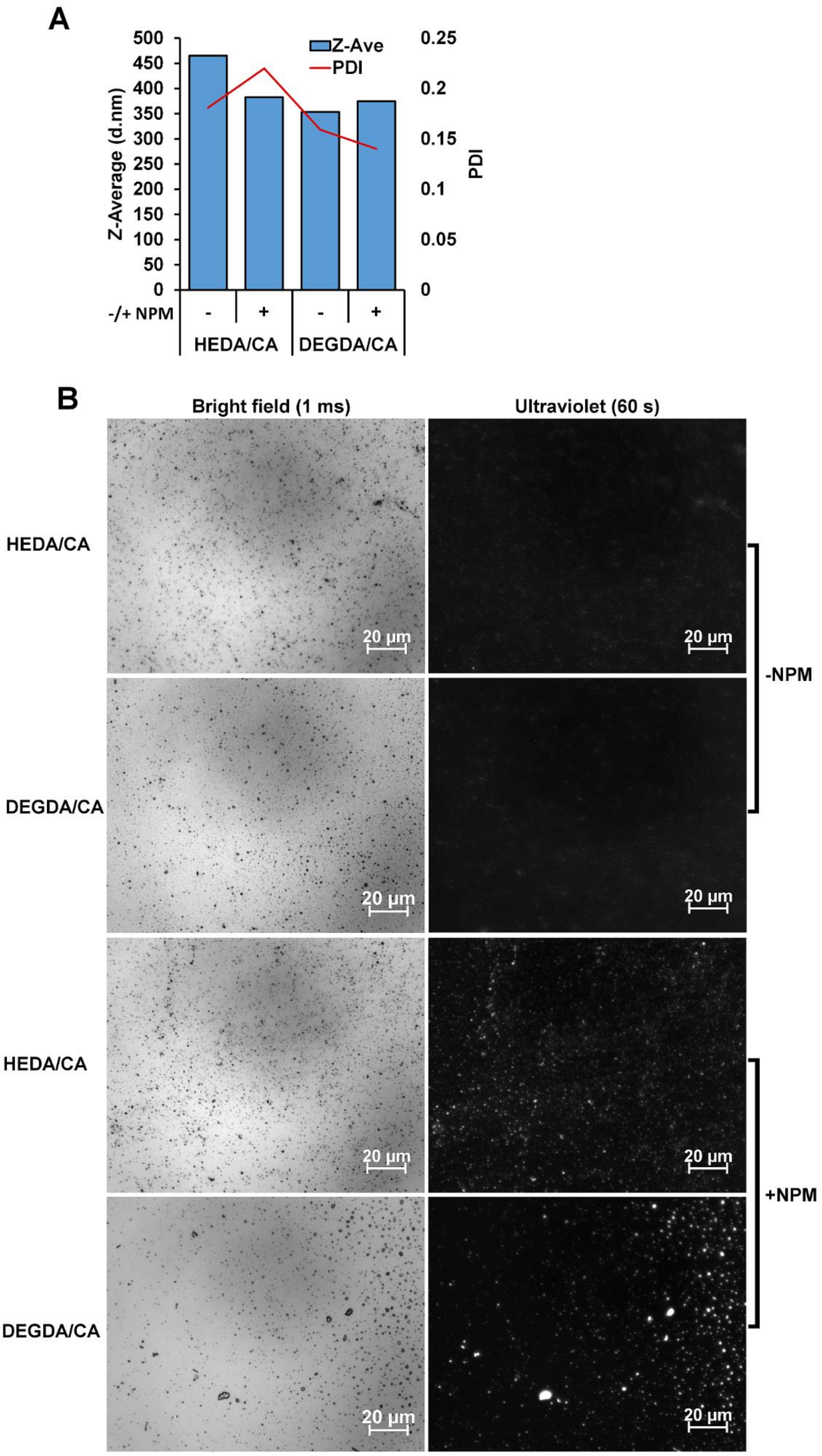
A
Z-Ave
PDI
Z-Average (d.nm)
PDI
-/+ NPM
HEDA/CA
DEGDA/CA
B
Bright field (1 ms)
Ultraviolet (60 s)
HEDA/CA
DEGDA/CA
HEDA/CA
DEGDA/CA
-NPM
+NPM
20 µm

Figure S3. Particle conjugation with NPM. A**)** Addition of *N*-(1-pyrenyl)maleimide (NPM) (ex/em 338/375 nm) to reduced nanoparticles in DI water. 1 hr incubation at room temperature with 50% DMSO, washed 3x. n=1 each. B**)** comparison of HEDA/CA and DEGDA/CA nanoparticles formed in DI water and added to 50 vol% DMSO ± NPM fluorescent maleimide. Particle aggregates appeared due to increased DMSO concentration, allowing optical detection of NPM.
